# Evaluation of the effect of different core substrates on the accuracy of intraoral scanners

**DOI:** 10.1002/cre2.899

**Published:** 2024-05-16

**Authors:** Maryam Khoshkhahesh, Shabnam Enteghad, Kiana Aghasadeghi, Mitra Farzin, Masumeh Taghva, Seyed Ali Mosadad

**Affiliations:** ^1^ Department of Prosthodontics, School of Dentistry Shiraz University of Medical Sciences Shiraz Iran; ^2^ Department of Oral Health Sciences, Faculty of Dentistry University of British Columbia Vancouver British Columbia Canada; ^3^ Student Research Committee Islamic Azad University of Shiraz Shiraz Iran; ^4^ Department of Research Analytics, Saveetha Dental College and Hospitals, Saveetha Institute of Medical and Technical Sciences Saveetha University Chennai India; ^5^ Department of Conservative Dentistry and Bucofacial Prostheses, Faculty of Odontology University Complutense of Madrid Madrid Spain

**Keywords:** dental impression technique, dimensional measurement accuracy, three‐dimensional imaging

## Abstract

**Background:**

The aim of this study was to determine if different types of core substrates have any effect on the trueness and precision of digital intraoral impressions.

**Material and Methods:**

A customized typodont with four similar cores of natural dentine, composite, metal (Ni‐Cr), and zirconia in the position of premolars was fabricated. The study model was scanned five times with two types of intraoral scanners (Carestream 3600 and 3Shape Trios 3), and a reference standard scan was obtained using a laboratory scanner (3shape D1000). A metrology software (Geomagic X) was used to align the data of experimental scans and the reference scan to determine deviation values (trueness). Precision values were calculated with random superimposition in each intraoral scanner group. The Kruskal–Wallis test was used to compare differences between different substrates, and the Mann–Whitney test was used to compare the average values between the two scanners.

**Results:**

Trios 3 was found to be significantly truer and more precise than Carestream 3600 (*p* value = .005, <0.001). There were no significant differences in the trueness of different substrates when they were scanned by Trios 3, while different materials showed significantly different trueness values in the Carestream 3600 group (*p* value = .003). Dentin showed the best trueness, and zirconia performed worse than other substrates. Regarding the precision of the scanners, neither of the scanners was affected by the type of scanning substrate.

**Conclusion:**

For Carestream 3600, substrate type did impact the trueness of intraoral scans, with dentin and zirconia showing the highest and lowest accuracy, respectively, while Trios 3 was similarly accurate across all substrates. Trios 3 had both higher trueness and precision than Carestream 3600.

## 1 INTRODUCTION

In many dental specialties, dental impressions are required for diagnostic purposes, treatment planning, and prosthetic and appliance production. The impression must precisely depict the patient's oral tissues to be useful. Inaccurate impressions lead to prosthesis misfit, which causes cement dissolution, prosthesis loss, cavities, and imprecise occlusion and articulation (Abduo & Elseyoufi, 2018).

The conventional impression methods have been the standard of care for many years, but they come with problems such as material preparation, continuous expenses, technical time, probable patient discomfort, and requirements of high clinical skills (Kravitz et al., 2014). Regardless of the material and technique, the conventional impression is inevitably flawed to some extent because it involves several steps in the clinic and laboratory that each could cause a degree of error in prosthesis fabrication (Ting‐shu & Jian, 2015). To address challenges with conventional procedures, digital impressions with intraoral scanner (IOS) and computer‐aided design and manufacturing (CAD/CAM) technologies were introduced to dental practice and have become increasingly popular for producing dental prostheses and models (Richert et al., 2017). The digital workflow was developed to make tooth restoration procedures quicker, more convenient, and more precise, with the aim of creating high‐strength and natural‐looking restorations (Ahlholm et al., 2018; Davidowitz & Kotick, 2011).

At present, IOSs are extensively used in dentistry for diagnosis, treatment planning, and fabricating restorations and devices. This includes not only their application in prosthodontics for manufacturing various fixed and removable prostheses like single crowns, fixed partial dentures, complete dentures, and mouthguards, but also their use in implantology for guided surgery and in orthodontics (Kihara et al., 2020; Mangano et al., 2017). However, intraoral scanners are not without limitations. These devices have been shown to be sensitive to intraoral fluids and scanning techniques, and factors such as operator experience, scan size, and variation in scanning substrate may impact the accuracy of data acquisition by IOSs (Renne et al., 2017; Resende et al., 2021).

It is clinically important that IOS devices be accurate. To describe the accuracy of a measurement method, the International Organization for Standardization (ISO) 5725 uses two terms: “trueness” and “precision.” “Trueness” is defined as the closeness of the arithmetic mean of test results to the true reference value, while “precision” refers to the closeness of agreement between test results (ISO, 1994).

The accuracy of intraoral scanning cannot be fully understood without considering the scanned substrate. Various post‐and‐core materials can be used to restore the damaged coronal portion of teeth after endodontic treatment to protect the teeth from fracture in the long term (Assif & Gorfil, 1994). Considering the accuracy of IOSs, the optical characteristics of the scanned substrate are important since the reflectivity, refractive index, and translucency (RR&T) of the substrate can alter the amount of light and the deflection path of light received by the sensor of an IOS, which can affect the quality of the 3D data captured by the sensor (Richert et al., 2017). Not only different restorative materials but also different dental tissues have variable RR&T (Meng et al., 2009). It has been demonstrated that even natural dentin can vary in refractive index depending on how the tubules are oriented in the scan area (Hariri et al., 2012). If RR&T is not taken into account in the design of an IOS system, disparities in light refraction among different substrates can reduce scan accuracy (Park et al., 2016).

While the majority of the literature on scanning accuracy contains comparisons between different IOS devices and systems, there are limited studies that have evaluated different substrates and their effect on the accuracy of the scanning process. These studies have shown that substrate type could affect the trueness and precision of an intraoral scan (Bocklet et al., 2019; Dutton et al., 2019; Elter et al., 2021; Lim et al., 2021). However, the results are inconsistent across different scanners and substrates. Furthermore, most studies have not considered the effect of morphology and position of teeth, which have been demonstrated to affect the accuracy of a digital scan (Son & Lee, 2020).

The aim of this study was to evaluate the effect of different dental core materials on the accuracy of two widely used intraoral scanners; Trios 3 and Carestream 3600. The following null hypotheses were addressed: (1) No significant differences exist between the accuracy of the two IOSs; (2) Substrate type would not affect the accuracy of the digital scan.

## 2 METHODS AND MATERIAL

The study design was approved by the ethical committee of Shiraz University of Medical Sciences (IR.SUMS.DENTAL.REC.1399.170). This study assessed the effect of various core substrates (natural dentin, metal, composite, and zirconia) on the accuracy of two intraoral scanners (CS 3600; Carestream Dental, Stuttgart, Germany, and TRIOS 3; 3Shape, Copenhagen, Denmark).

### 2.1 Study model preparation

A maxillary premolar was prepared for a full ceramic crown, ensuring that the whole preparation was placed in dentin. This tooth was used as a standard core model. Using a desktop scanner (3shape D1000; 3Shape), a scan of the prepared tooth was performed; according to the generated standard tessellation language (STL) file, similar cores of composite (laboratory indirect composite resin; Gradia, GC Corp.), Ni‐Cr alloy (4all; Ivoclar Vivadent), and zirconia (pre‐sintered Y‐TZP monolithic highly translucent zirconia; DD Bio ZX2; Dental Direkt) were designed and milled (inLab MC XL; Dentsply Sirona). This method was used to ensure that all cores had identical shapes and dimensions. Afterward, using epoxy resin, the four produced cores were fixed in a typodont model in the position of premolars.

### 2.2 Reference scan fabrication

The study model was directly scanned using a laboratory scanner (3Shape D1000; 3Shape) to obtain the reference scan data. The accuracy of this scanner has been confirmed to be as high as 5 µm, according to the ISO 12836 standard. Additionally, it has demonstrated considerably high precision, similar to the ATOS industrial scanner, averaging at 0.5 µm, which is significantly higher than the precision of intraoral scanners (Nedelcu et al., 2018).

### 2.3 Experimental scan fabrication

The study model was scanned five times by each of the two intraoral scanners (CS 3600 and TRIOS 3). Since the accuracy of scanning systems could be influenced by operator experience (Resende et al., 2021; Waldecker et al., 2021), the experimental scans were carried out by specific operators with advanced experience on each IOS system. Each operator followed the scan patterns and protocols recommended by the manufacturer. Table 1 contains details regarding the scanning systems used in this research.

**Table 1:** Intraoral scanning systems used in the study.

Product name	Manufacturer	Scanner technology	Acquisition method	Powder use	Software version
TRIOS 3	3shape	Confocal microscopy	Ultrafast optical sectioning technique	Not required	Trios software v. 1.18.2.6
Carestream 3600	Carestream Dental	Triangulation	continuous video image capture system Active Speed 3D Video™	Not required	CS IO 3D acquisition software, v. 3.1.0

### 2.4 Three‐dimensional analysis

The associated program exported each test scan in STL format. To evaluate accuracy (trueness and precision), a 3D inspection and metrology program (Geomagic Control X; 3D Systems) was employed. The reference standard scan data were initially trimmed within 1 mm from the gingival margin to remove extra data points on the cast base, keeping only the teeth aligned. The eliminated data points from the reference standard scan were prevented from being compared with the IOS scan files.

For evaluating the trueness of the IOSs, experimental scan files were imported and then superimposed onto the reference scan, using an Initial Alignment and a Best Fit Alignment algorithm considering only one of the four core substrates on the typodont. To align the test file and reference file, the Best Fit Alignment tool of the software uses an iterative closest point algorithm, a standard technique for aligning digital 3D data. Following alignment, the 3D Compare function enabled the separation of substrate regions from the dental arch to calculate the deviation of all points of interest (POI) of a particular substrate. Extra parts beyond the margin of prepared cores were removed to measure only the difference in the preparation area. After that, color maps were created, and all deviations were calculated and visualized. The upper and lower color mapping limits were established at ±500 µm. To show deviations between superimposed structures, inward and outward displacements were represented by blue and red colors, respectively, while green color indicated no change. Data reports, including deviation mean, maximum, minimum, and standard deviation, were created for each comparison. For evaluating precision, the scan data in each scanner group were randomly superimposed in a pairwise manner (five times per group).

### 2.5 Statistical analysis

The mean and standard deviation values of trueness and precision were calculated for each set of scans. Statistical analysis was performed using the Shapiro–Wilk test to assess data distribution. Kruskal–Wallis and Tocky post hoc tests were used to compare the average values of trueness and precision obtained from scanners between different types of materials, and the Mann–Whitney test was used to compare the average values between the two scanners. All data were analyzed using SPSS 26 (IBM Corporation), and the statistical significance level was set at .05.

## 3 RESULTS

The Mann–Whitney test showed a significant difference between Trios 3 and CS 3600 scanners with regard to precision (*p* < .001), where Trios 3 showed better precision (lower values) (Table 2). A lower precision value corresponds to a smaller average deviation from other scans made with the same scanner. Similarly, in trueness analysis, Trios 3 showed significantly better trueness than CS 3600 (*p* = .005) (Table 3). Greater trueness is indicated by a lower mean trueness value which means less average deviation of test scans from the reference.

**Table 2:** Statistical analysis on the precision of scanners using summed data from all materials (All values are in µm).

Scanner	Mean ± SD	Std. Error	Min	Max	95% Confidence Interval for mean		*p* Value
					Lower bound	Upper bound	
Trios 3	8.02 ± 4.59	1.03	1.74	20.11	5.87	10.17	<.001*
CS 3600	20.47 ± 7.44	1.66	7.50	33.30	16.99	23.96	

*Note*: Mann–Whitney test was used.

Abbreviations: Min, minimum; Max, maximum; SD, standard deviation; Std. Error, standard error.

* *p* ≤ .05; number of repetition = 5.

**Table 3:** Statistical analysis on the trueness of scanners using summed data from all materials (All values are in µm).

Material	Mean ± SD	Std. Error	Min	Max	95% Confidence Interval for Mean		*p* Value
					Lower bound	Upper bound	
Trios 3	25.85 ± 10.31	2.31	12.10	50.64	21.02	30.68	.005*
CS 3600	42.39 ± 21.37	4.78	18.58	109.16	32.32	52.33	

*Note*: Mann–Whitney test was used.

Abbreviations: Min, minimum; Max, maximum; SD, standard deviation; Std. Error, standard error.

* *p* ≤ .05.

Summary statistics for the precision and trueness of each substrate in the two scanner groups are presented in Tables 4 and 5, respectively. For both scanners, dentin showed the best trueness and precision among all materials, and zirconia performed worse than other substrates, including metal, composite, and dentin. Regarding precision, the Kruskal–Wallis test revealed that the differences between the values for each substrate were not statistically significant in Trios 3 (*p* = .127) and CS 3600 (*p* = .075) groups. In contrast, statistical analysis for comparison of mean trueness values of substrates showed a significant difference between different substrates in the CS 3600 group (*p* = .003). However, the trueness values did not differ significantly between different substrates scanned by Trios 3 (*p* = .099).

**Table 4:** The mean and standard deviation values of precision (µm) by material and scanner.

Scanner	Dentin (*n* = 5)	Composite (*n* = 5)	Metal (*n* = 5)	Zirconia (*n* = 5)	*p* Value
Trios 3	4.7 ± 1.8	7.3 ± 2.5	9.4 ± 2.8	11.0 ± 6.2	.127
CS 3600	15.5 ± 3.9	19.4 ± 6.6	19.7 ± 7.0	27.3 ± 7.9	.075

*Note*: Data are presented as mean ± SD.

Abbreviation: SD, standard deviation.

**Table 5:** The mean values of trueness (µm) by material and scanner.

Scanner	Material				
	Dentin (*n* = 5)	Composite (*n* = 5)	Metal (*n* = 5)	Zirconia (*n* = 5)	*p* Value
Trios 3	18.1 ± 2.2	23.4 ± 6.8	26.9 ± 8.3	35.0 ± 13.9	.099
CS 3600	21.0 ± 2.5	38.3 ± 10.6	45.3 ± 12.3	65.0 ± 25.7	.003*

*Note*: Data are presented as mean ± SD.

Abbreviation: SD, standard deviation.

The pairwise comparison of individual substrate mean trueness values in the CS 3600 group, using Tukey's HSD post hoc test, revealed that the trueness of dentin was significantly better than that of zirconia (*p* = .002) (Table 6).

**Table 6:** Pairwise comparisons of materials for CS 3600 scanner (all values are in µm).

Materials		Mean difference	Std. Error	*p* Value
Dentin	Composite	−17.30	9.63	.31
	Metal	−24.27	9.63	.094
	Zirconia	−44.00	9.63	.002*
Composite	Metal	−6.97	9.63	.89
	Zirconia	−26.7	9.63	.059
Metal	Zirconia	−19.72	9.63	.21

*Note*: Tukey's HSD posthoc test was used.

Abbreviation: Std. Error, standard error.

* *p* < .05.

List of Abbreviations used in this study is in Table 7.

**Table 7:** List of abbreviations.

Abbreviation	Definition
IOS	Intraoral scanner
CAD/CAM	Computer‐aided design and manufacturing
CS 3600	Carestream 3600
RR&T	Reflectivity, refractive index, and translucency
STL	Standard tessellation language
SD	Standard deviation
Std. Error	Standard error

## 4 DISCUSSION

The aim of this study was to assess the trueness and precision of scans of different core substrates with two widely used intraoral scanners, Trios 3 and Carestream 3600. A high‐accuracy desktop scanner (3Shape D1000) was used to obtain standard reference scans as in previous studies (Resende et al., 2021; Revilla‐León et al., 2022; Zhang et al., 2022). For the most accurate results in in vitro research, preparing specimens using a standard protocol is essential. Researchers have discovered that the tooth geometry and its placement may have an impact on the accuracy of IOS systems (Keeling et al., 2017; Mejía et al., 2017). In this study, CAD/CAM technology was employed for the preparation of core specimens to obtain the most identical samples, and they were all placed in the position of premolars to eliminate potential bias from the effect of tooth position in the dental arch on the accuracy of the scan.

In this study, the values for different substrates were pooled to evaluate the trueness and precision of the different intraoral scanners. Trios 3 showed both higher precision (8.02 vs. 20.47 μm) and trueness (35.85 vs. 42.39 μm) than CS 3600; therefore, the first null hypothesis was rejected. Regarding the trueness of scanners, in the study of Bocklet et al. (2019), on the effect of scan substrate on the accuracy of digital scans, the trueness values for all examined substrates were higher in the CS 3600 group than in the Trios 3 group. These findings are in accordance with those of the present study. Other studies evaluating the level of accuracy on the single tooth (Pellitteri et al., 2022; Zimmermann et al., 2020) and complete arch (Michelinakis et al., 2022) have also shown that Trios 3 was significantly truer than CS 3600. However, contrary to our findings and the aforementioned studies, several studies on the accuracy of full‐arch or partial‐arch dental scans have reported no statistically significant difference in trueness between TRIOS 3 and CS 3600 (Ender et al., 2019; Nulty, 2021; Viegas et al., 2021; Winkler & Gkantidis, 2020).

Concerning the precision of IOSs, in our study, Trios 3 was found to be more precise than the CS 3600 scanner. This result confirms the findings of several other studies that have compared these two scanners and reported statistically higher local and full arch precision for Trios 3 (Michelinakis et al., 2022; Viegas et al., 2021; Winkler & Gkantidis, 2020; Zimmermann et al., 2020). However, there are reports of similar complete arch precision of these intraoral scanners in the literature (Ender et al., 2019; Nulty, 2021). Despite major differences between studies in terms of study design, 3D difference evaluation methods (percentiles, root mean square, etc.), number of teeth analyzed, the analysis software used, and methods for obtaining reference scans, it appears that Trios 3 has similar or higher accuracy compared to CS 3600. The differences in the accuracy of IOSs can be attributed to the different acquisition techniques, scanning protocols, and software algorithms for post‐processing the 3D model used by these intraoral scanners.

The second null hypothesis was partially rejected as the core substrate impacted the accuracy of CS 3600 but did not have an effect on that of Trios 3. For CS 3600, the trueness values ranged from 21 to 65 μm, and zirconia performed significantly worse than dentin. This is in accordance with the study of Bocklet et al. (2019), where substrate impacted the trueness values of scans made by this scanner, ranging from 23.2 to 46.9 μm. According to this study, for CS 3600, dentin displayed higher trueness than all other substrates (amalgam, composite, and enamel) and all substrates displayed higher trueness than enamel. Furthermore, dentin was either the truest or co‐truest material for the majority of scanners examined in this study. It can be assumed that, compared to other substrates, dentin's physical characteristics allow for more accurate scanning. As evidence in the literature suggests, higher translucency substrates may cause intraoral scanners to operate less accurately. Dutton et al. (2019) showed that for many scanners (Medit i500, iTero element, Primescan, and Emerald), the more translucent substrates such as natural enamel, enamel shade composite, and lithium disilicate had a negative influence on both trueness and precision and interestingly, highly reflective substrates like polished metals did not show the same effect, which is consistent with the findings of the present study. Li et al. (2017) evaluated how the accuracy of a powder‐free IOS (Organical, R + K CAD/CAM Technology) was affected by the translucency of ceramic copings, and their findings are in accordance with those of the current study and the studies mentioned previously. They reported that materials with higher translucency were scanned with less accuracy and more morphological alterations. In another study (Elter et al., 2021) to evaluate the trueness of digital scans of different composite resin cores, it was observed that universal composite resins displayed higher trueness than flowable and bulk fill composites. This finding was attributed to the lower translucency of universal composites compared to other composite materials.

For Trios 3, the trueness values of different substrates ranged between 18.1 and 35 μm, and a similar trend was observed where dentine showed the highest trueness followed by composite, metal, and zirconia, respectively. However, there were not any statistically significant differences between the trueness values of different materials, indicating that substrate type did not have an effect on the trueness of this scanner. Trios 3 employs confocal microscopy technology, while the image acquisition principle for CS 3600 is active triangulation. Dutton et al. (Dutton et al., 2019) have reported that confocal microscopy IOS devices seem to be less sensitive to substrate variations compared to active triangulation scanners. In their study, Trios 3 was the only scanner, the trueness of which was not influenced by the type of substrate. The trueness values in this study ranged between 15.2 and 33 μm for different restorative materials, which is in agreement with our results. In contrast, in the study of Bocklet et al., substrate did impact the trueness values in Trios 3 (ranging from 20.9 to 34.1 μm), where the trueness of scans of enamel was significantly lower than those of other substrates (amalgam, composite, and dentin) and composite was truer than amalgam. Michelinakis et al. (2022) also reported that substrate type had a significant effect on the trueness of both CS 3600 and Trios 3, and they reported a higher range of 37.2 to 116.6 μm. These discrepancies can be due to differences in study designs, the type of restorative materials, and the position and geometry of the scanning substrate, which make the comparison of the results of this study to previous literature difficult. Nevertheless, the mean trueness of both examined IOSs does not exceed the acceptable limit of 100 μm. However, it is still uncertain what level of deviation for a digital intraoral impression is acceptable for successful treatments in a clinical environment (Gassino et al., 2004).

In the present study, substrate type did not influence the precision of either of the tested scanners. In accordance with our results, Bocklet et al. (2019) reported no differences in the precision of different substrates for both Trios 3 and CS 3600. In fact, among the seven tested IOSs, only the precision of Emerald was impacted by the type of substrate, where scans of enamel were found to be more precise compared to composite. Dutton et al. (2019) also showed that for Trios 3, substrate type did not have an effect on the scanning precision.

A limitation of the present study was the limited substrates. In this study, only one metal alloy, and one type of composite and zirconia were compared. Further studies are recommended to investigate substrates such as different metals, ceramics, and composites with various shades and translucencies. Another drawback of the current study may be that the reference scanner, despite being quite powerful, was a laboratory scanner. It might be better to utilize a stronger optical industrial scanner, or preferably, a contact scanner, such as an articulated arm or a coordinate measuring machine, which has the ability to physically probe the object surface being scanned.

It is suggested to consider both the material and the surface optical properties (roughness criteria) to conduct a more relevant study. Finally, this study had an in vitro design. Clinical confounding variables, such as patient movement, saliva, blood, and accessibility restrictions, were not examined. Hence, in vivo research is required to confirm the findings of the present study.

## 5 CONCLUSION

Within the limitations of this study, it was concluded that Trios 3 was statistically truer and more precise than the Carestream 3600 scanner. For Carestream 3600, substrate type did impact the trueness of intraoral scans, with dentin being the most accurate and zirconia being the least accurate; Trios 3 was similarly accurate across all substrates.

## AUTHOR CONTRIBUTIONS

Maryam Khoshkhahesh and Shabnam Enteghad developed the theory and performed the computations. Kiana Aghasadeghi and Mitra Farzin verified the analytical methods. Masumeh Taghva and Seyed Ali Mosadad supervised the findings of this work. All authors discussed the results and contributed to the final manuscript.

## CONFLICTS OF INTEREST STATEMENT

The authors declare no conflict of interest.

## Data Availability

Data are available upon the request of readers.
